# The requirement of glycoprotein C (gC) for interindividual spread is a conserved function of gC for avian herpesviruses

**DOI:** 10.1038/s41598-021-87400-x

**Published:** 2021-04-08

**Authors:** Widaliz Vega-Rodriguez, Huai Xu, Nagendraprabhu Ponnuraj, Haji Akbar, Taejoong Kim, Keith William Jarosinski

**Affiliations:** 1 Department of Pathobiology, College of Veterinary Medicine, University of Illinois at Urbana-Champaign, Urbana, IL USA; 2 United States Department of Agriculture, Agricultural Research Service, US National Poultry Research Center, Athens, GA USA

**Keywords:** Vaccines, Virology

## Abstract

We have formerly shown that glycoprotein C (gC) of *Gallid alphaherpesvirus* 2, better known as Marek’s disease (MD) alphaherpesvirus (MDV), is required for interindividual spread in chickens. Since gC is conserved within the *Alphaherpesvirinae* subfamily, we hypothesized gC was important for interindividual spread of other alphaherpesviruses. To test this hypothesis, we first generated a fluorescent protein tagged clone of *Gallid alphaherpesvirus* 3 MD vaccine strain 301B/1 to track virus replication in cell culture and chickens using fluorescent microscopy. Following validation of this system, we removed the open reading frame of 301B/1 gC from the genome and determined whether it was required for interindividual spread using experimental and natural infection studies. Interindividual spread of MD vaccine 301B/1 was abrogated by removal of 301B/1 gC. Rescuent virus in which 301B/1 gC was inserted back into the genome efficiently spread among chickens. To further study the conserved function of gC, we replaced 301B/1 gC with MDV gC and this virus also efficiently spread in chickens. These data suggest the essential function of alphaherpesvirus gC proteins is conserved and can be exploited during the generation of future vaccines against MD that affects the poultry industry worldwide.

## Introduction

Herpesviruses have co-evolved with their respective hosts for millions of years. In most cases, each herpesvirus and host have reached a relatively stable relationship with many hosts infected with multiple herpesviruses, including humans that currently have nine associated herpesviruses^1^. Although there is a tremendous amount of information on herpesvirus-host interactions in cell culture, little is known about their relationships pertaining to interindividual spread and the viral and cellular genes that mediate this important aspect of the virus lifecycle. This is primarily due to the difficulty of studying the mechanistic nature of human herpesviruses in humans, as well as a lack of many natural animal models. For this, we turn to natural herpesvirus-host models to address questions on interindividual spread and dissemination in populations.

For much of the 1990s, there was a tremendous amount of attention paid to conserved herpesvirus glycoprotein C (gC) homologues due to their high expression levels and immunogenicity, where they were shown to perform multiple functions in vitro*.* Some of the functions identified include primary attachment of cell-free virus to heparin sulfate (HS)- and chondroitin-like glycosaminoglycans (GAGs) on the surface of cells^2,3^, and involvement in late steps of virus egress from cultured cells^2,4^. Although gC is not essential for most herpesviruses studied thus far, it significantly increases the efficiency of infection by providing an additional binding mechanism^5^ and helps shield the virus from antibody neutralization^6^. In addition to viral attachment and egress, gC homologues are thought to have immune evasion functions mediated by binding to and inhibiting the action of complement component C3^7–12^, as well as a role in chemokine-mediated leukocyte migration^13^. Similar to MDV, gC homologues for herpes simplex virus 1 (HSV-1) and varicella-zoster virus (VZV) appear to play a minor role in tissue culture model systems but are critical for HSV-1 and VZV replication in human skin cells using the SCID-hu mouse model^14^ suggesting gC homologs may perform conserved functions during natural infection of the host. However, studies on the role of gC homologs are limited due to a lack of natural animal host model systems.

We have formerly shown that gC of Marek’s disease alphaherpesvirus (MDV) is dispensable for in vitro and in vivo replication but is required for interindividual spread from chicken to chicken^15–18^. MDV causes Marek’s disease in chickens, presenting with severe clinical symptoms including the development of solid lymphomas in the viscera and other organs; metabolic dysfunction; and neurological signs like paralysis and ataxia^19^. It is a major economic problem in the poultry industry due to its global distribution and transmissibility^20^. Natural infection of MDV begins through the respiratory route by inhalation of infectious virus where pulmonary B lymphocytes and macrophages or dendritic cells^21^ are initially infected and transport the virus to lymphoid organs. Primary cytolytic infection ensues in activated T lymphocytes recruited to the sites of infection, which become the primary cell type infected, and latency is established in these cells. Depending on the line of chicken or MDV strain, oncogenic transformation of latently infected T cells results in lymphoma formation that is ultimately a dead-end for the virus. Important for dissemination in the population, migrating infected cells transport MDV to feather follicle (FF) epithelial (FFE) cells in the skin, where infectious cell-free virus is shed into the environment, and the virus life cycle can repeat in new hosts.

There are currently eight herpesviruses identified in avian species with all characterized within the subfamily *Alphaherpesvirinae* in the *Herpesviridae* family^1^. Of the eight avian herpesviruses, six belong to the *Mardivirus* genus of which MDV or *Gallid alphaherpesvirus* 2 (GaHV-2) is the prototypic virus within this genus. MD is controlled through vaccination with attenuated MDV strains and homologous non-oncogenic avian herpesviruses, including *Gallid alphaherpesvirus* 3 (GaHV-3) and turkey herpesvirus (HVT: *Meleagrid alphaherpesvirus*; MeHV-1). However, the current vaccines are efficient at reducing tumor formation and disease but not block interindividual spread of virulent MDV resulting in increasing virulence over the decades^22^. It is generally accepted that GaHV-3 and HVT have similar interindividual spread pathogeneses as MDV.

Here, we hypothesized that the absolute requirement of gC for MDV interindividual spread is conserved among other avian herpesviruses. To test this hypothesis, we used a recently generated infectious bacterial artificial chromosome (BAC) clone of the MD vaccine strain 301B/1^23^ in experimental and natural infections of chickens to determine whether 301B/1 gC is required for 301B/1 transmission. Our results conclusively showed that 301B/1 gC is required for interindividual spread and that MDV gC could compensate for 301B1 gC in this process. These results suggest the importance of gC homologs in interindividual spread may be a conserved function and draws importance to studying this glycoprotein during interindividual spread of other herpesviruses.

## Results

### Generation of recombinant (r)301B/1 expressing pUL47mRFP (r301B47R)

**Figure 1:**
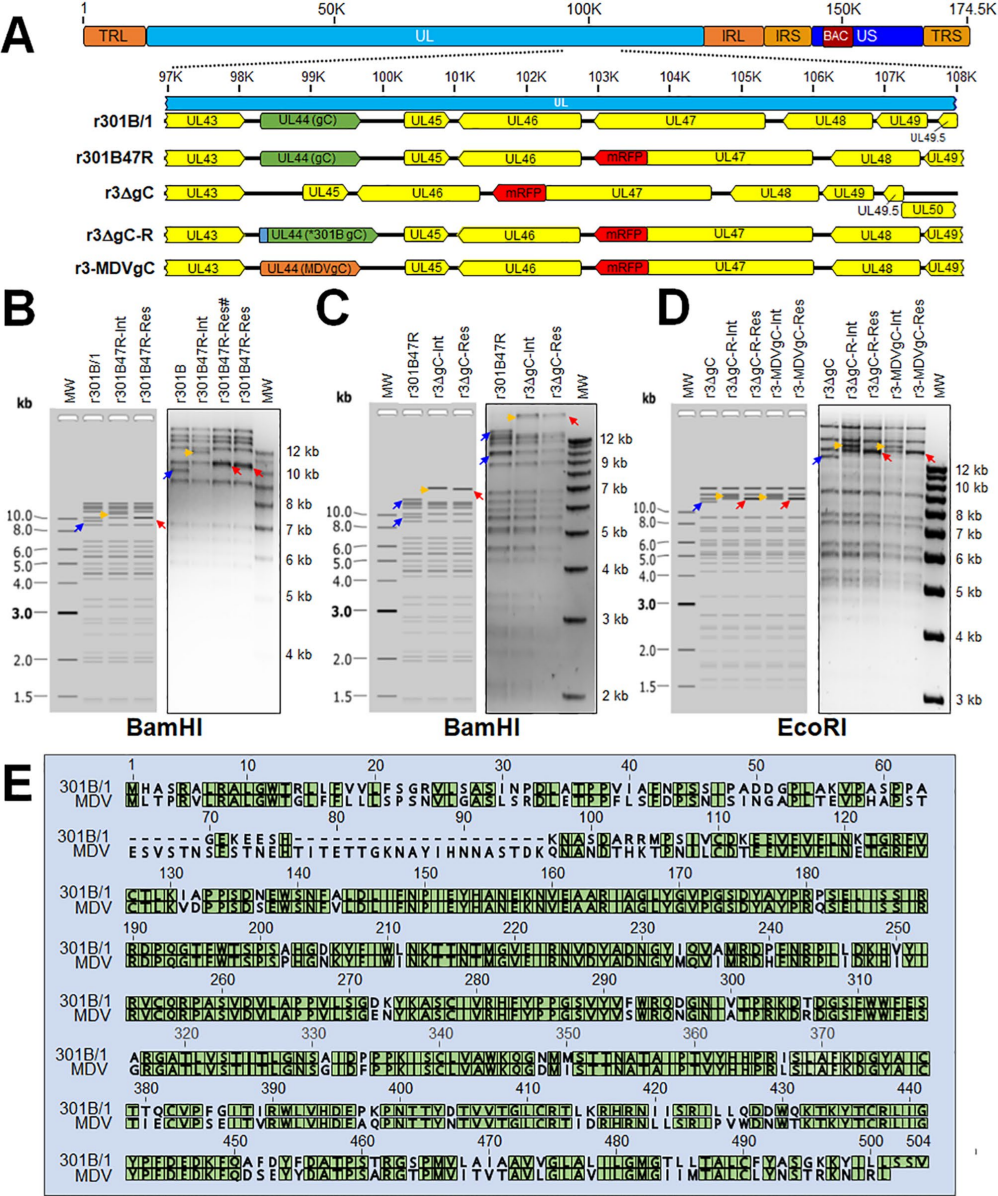
Generation of r301B/1clones. (**A**) Schematic representation of the 301B infectious clone^23^ genome depicting the locations of the terminal repeat long (TRL) and short (TRS), internal repeat long (IRL) and short (IRS), and unique long (UL) and short (US) regions. The region of the UL spanning UL43 to UL50 is expanded to show the relevant genes within this region and modifications for each r301B/1 clone. (**B**) Predicted and actual RFLP analysis of r301B/1 clones. BAC DNA obtained for r301B, r301B47R-integrate clone and two resolved clones were digested with BamHI and electrophoresed through a 1.0% agarose gel. Integration of the mRFP + *AphAI* sequence resulted in an increase in the 9545 bp (blue colour rightwards arrow) fragment to 11,207 bp (yellow colour rightwards arrow). Resolution by removal of the *AphAI* sequence shifted the 11,207 bp fragment to 10,233 bp (red colour leftwards arrow). One resolved clone (#) was used after this point. (**C**) Predicted and actual RFLP analysis of r3ΔgC r301B clone. BAC DNA obtained for r301B47R, r3ΔgC-integrate clone and r3ΔgC-resolved clone were digested with BamHI and electrophoresed through a 1.0% agarose gel. Integration of the *AphAI* sequence into this locus removed a BamHI site combining the 10,223 and 14,854 bp fragments (blue colour rightwards arrow) to 24,671 bp (yellow colour rightwards arrow). Resolution of the *AphAI* sequence reduced the 23,933 bp fragment by 1028 bp to 23,643 bp (red colour leftwards arrow). (**D**) Predicted and actual RFLP analysis of r3ΔgC-R and r3-MDVgC clones derived from the r3ΔgC clone. BAC DNA obtained for r3ΔgC, r3ΔgC-R-integrate, r3ΔgC-R-resolved, r3-MDVgC-integrate, and r3-MDVgC-resolved clones were digested with EcoRI and electrophoresed through a 1.0% agarose gel. Integration of 3 × Flag301BgC-*AphAI* or MDVgC-*AphAI* sequences into this locus resulted an increase in the 12,879 bp (blue colour rightwards arrow) fragment to 15,420 bp (yellow colour rightwards arrow) or 15,403 bp (yellow colour rightwards arrow), respectively. Removal of the *AphAI* sequence from r3ΔgC-R-Int reduced the 15,420 bp fragment by 1038 bp to 14,382 bp (red colour leftwards arrow) to generate r3ΔgC-R-Res. Removal of the *AphAI* sequence from r3-MDVgC-Int reduced the 15,403 bp fragment by 1034 bp to 14,369 bp (red colour leftwards arrow) to generate r3-MDVgC-Res. The molecular weight marker was the 1 kb Plus DNA Ladder from Invitrogen, Inc. (Carlsbad, CA). No extraneous alterations are evident. (**E**) Alignment of 301B/1 and MDV (RB-1B strain) gC protein using MUSCLE Alignment in Geneious Prime 2021.0.3 (Biomatters, Inc., San Diego, CA). Green highlighted amino acids are conserved between the two proteins.

We and others have shown that fusing fluorescent proteins to the C-terminus of alphaherpesvirus pUL47 (VP13/14) allows the visualization of infected cells and does not affect replication in cell culture and in vivo for numerous herpesviruses^24–28^. Therefore, we inserted monomeric red fluorescent protein (mRFP) at the C-terminus of the pUL47 in a recently described BAC clone of 301B/1^23^ to generate r301B47R (Fig. 1A). RFLP analysis confirmed the integrity of the BAC clones as the predicted banding pattern was observed described in the figure (Fig. 1B). In addition, DNA sequencing was used to confirm that each clone was correct at the nucleotide level (data not shown) using primers specific for each gene (Table 1).

**Table 1:** Primers used for sequencing.

Gene^a^	Direction^b^	Sequence (5′ → 3′)
UL47cTerm	Forward	CCTTCTCGGCACGCTAGCCT
	Reverse	TTTTGGGACGCGAAGTGGCC
UL44 (gC)	Forward	GCTAAGTTGCGCAGGCAGAG
	Reverse	GGGCCGGATGTACCTATACG
	Forward	GACCCGCCTCGGTCGACG
	Reverse	ACATAGACGGAGCCCGGTGG
	Forward	GCCATCGACGAGGGGGT
	Reverse	GCCGGAATACTTGACGGGTTG

^a^Gene sequenced with the set of primers.

^b^Directionality of the primer.

### Replication of v301B47R in cell culture

**Figure 2:**
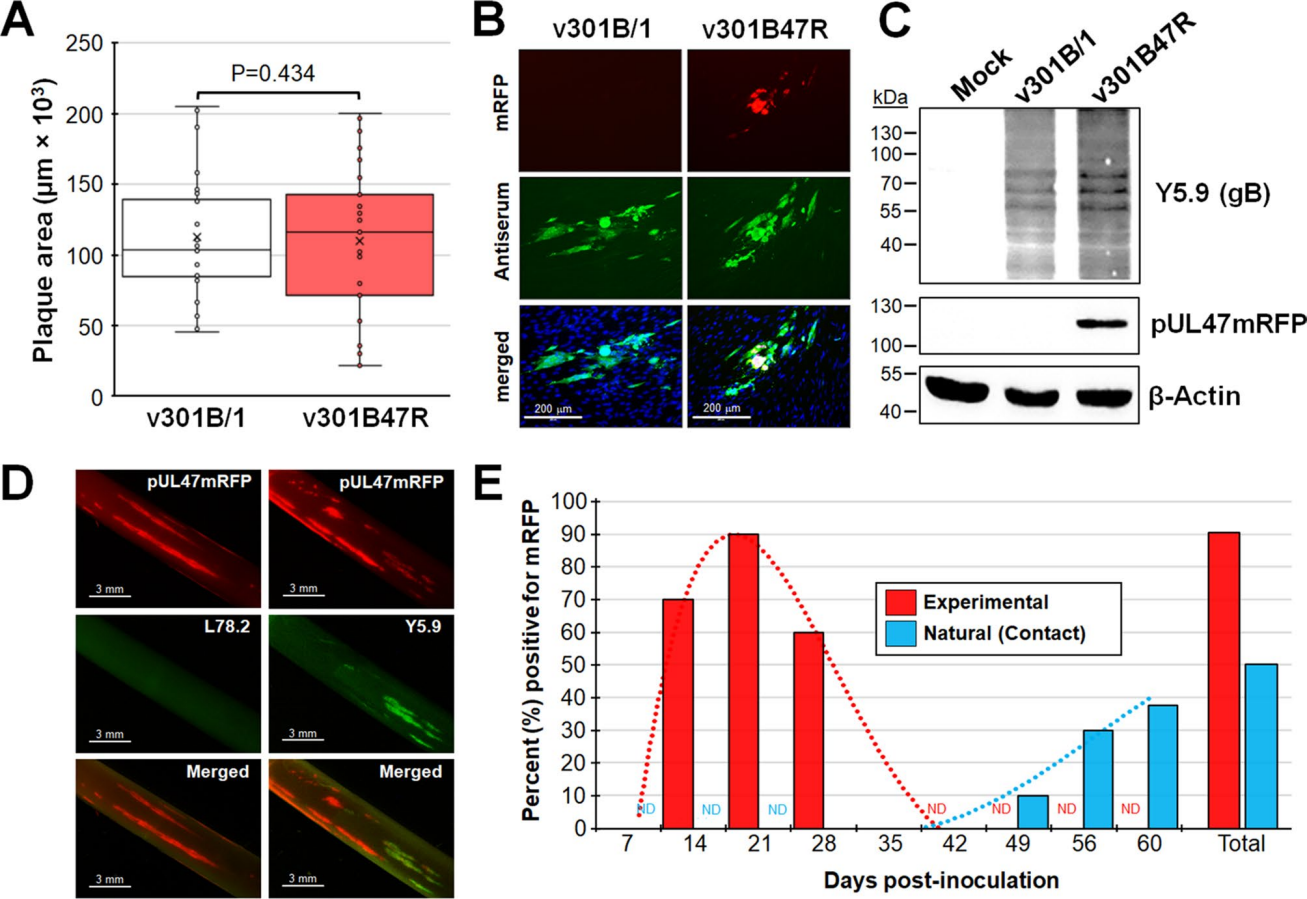
Replication and fluorescent protein expression in tissue culture cells. (**A**) Mean plaque areas (n = 25) of viruses reconstituted from r301B/1 and r301B47R were measured and the results are shown as box & whisker plots. There were no significant differences in plaque sizes between the two viruses using Student’s *t* tests. (**B**) Representative plaques for v301B/1 and v301B47R are shown. Plaques were stained with polyclonal chicken anti-GaHV-3 antibody and goat anti-chicken-IgY Alexa488 (green) was used as secondary antibody to identify plaques. Fluorescent expression of mRFP (red) was directly visualized and cells were counterstained with Hoechst 33342 to visualize nuclei. (**C**) Western blotting for pUL47mRFP using anti-mRFP antibody. The anti-GaHV-3 antibody Y5.9^31^ was used to show the relative level of infection in the cultures. Antibody against chicken β-actin is shown as a loading control. (**D**) Fluorescent protein expression in feather follicles (FFs) infected with v301B47R at 21 dpi. FFs were also stained with anti-HVT L78.2 or -GaHV-3 Y5.9 plus anti-mouse IgG-Alexa488 (green) and images were collected with a fluorescent stereoscope. (**E**) Percent of chickens positive for pUL47mRFP in experimental and naturally (contact) infected chickens over 60 days. *ND* not done.

Following reconstitution of r301B/1 and r301B47R resulting in v301B/1 and v301B47R, respectively, we first tested in vitro growth properties using plaque size assays. Consistent with fusing fluorescent proteins to pUL47 in other herpesviruses, there was no change in replication between parental v301B/1 and v301B47R during cell culture replication in chicken embryo cells (CEC) cultures (Fig. 2A). Additionally, while reconstituting r301B47R in DF-1-Cre (data not shown) and propagating in CEC cultures, mRFP was abundantly expressed that could be visualized using fluorescent microscopy (Fig. 2B). Expression of pUL47mRFP was almost exclusively found in the nucleus, consistent with MDV, though the expression levels appeared to more abundant than observed for MDV^25,29^. Western blotting using anti-mRFP antibody showed mRFP expression was fused to the pUL47 since mRFP alone is ~ 26 kDa in size, while fused to pUL47 would create ~ 115 kDa protein (Fig. 2C). These results show that fusing mRFP to the C-terminus of pUL47 of 301B/1 resulted in no change in viral replication in tissue culture and allowed the direct visualization of 301B/1 replication in cells. This data is consistent with former studies fusing fluorescent proteins to pUL47 homologs^25–27,30^.

### v301B47R as a tool for tracking virus in chickens

Next, we tested the ability of v301B47R to replicate and interindividual spread in chickens. To do this, ten chickens were experimentally infected with 4000 PFU v301B47R and housed with ten naïve contact chickens for nine weeks to measure natural infection (interindividual spread). First, we were interested in whether we could directly identify infected birds based on fluorescence in plucked feathers as has been previously done in our laboratory for MDV^32^. Like our former results with fluorescently tagged MDV, feather follicles were easily identified from feathers (Fig. 2D) in most experimentally infected chickens at 14 to 28 days post-infection (dpi) with a total of 90% of birds positive for v301B47R by 21 dpi (Fig. 2E). Staining of FFs showed feathers positive for pUL47mRFP were also positive for anti-GaHV-3 glycoprotein B (Y5.9), while negative for anti-HVT glycoprotein B (L78.2). These results show that fusing mRFP to 301B/1 pUL47 can be an effective tool to track 301B/1 in chickens.

### v301B47R can spread from chicken to chicken

Over the course of 9 weeks, 50% of the naïve contact chickens housed with experimentally infected chickens became positive by the time the experiment was terminated (Fig. 2E). There was a delay of about three weeks before naïve contact chickens began to show fluorescent feathers compared to experimentally infected chickens, which is consistent with the time it takes for MDV to interindividual spread. These results confirm that 301B/1 can interindividual spread from chicken to chicken.

### Generation of r301B/1 lacking gC or expressing 3 × Flag301B gC or MDV gC

Now that we had a tool to track 301B/1 in cell culture and chickens, we wanted to test two hypotheses. First, we hypothesized that 301B/1 gC, like MDV gC, would be required for interindividual spread in chickens. Second, we hypothesized that, since both GaHV-3 and MDV are chicken herpesviruses with similar pathogeneses, MDV gC would compensate for 301B/1 replication and transmission. Therefore, we removed the complete UL44 (gC) open reading frame (ORF) from r301B47R to generate r3ΔgC (Fig. 1A). To generate a rescuent virus, 301B/1 gC was inserted back into the viral genome where it was originally removed but included a 3 × Flag tag at the N-terminus (r3-ΔgC-R) that should allow us to identify 301B/1 gC in downstream studies. In addition, we inserted MDV gC in its place to generate r3-MDVgC. RFLP analysis confirmed the integrity of the BAC clones as the predicted banding pattern was observed (Fig. 1C, D). In addition, DNA sequencing confirmed that each clone was correct at the nucleotide level (data not shown) using primers specific for each gene (Table 1).

### Replication of v301B/1 lacking gC (v3ΔgC) or expressing MDV gC (v3-MDVgC) in cell culture

**Figure 3:**
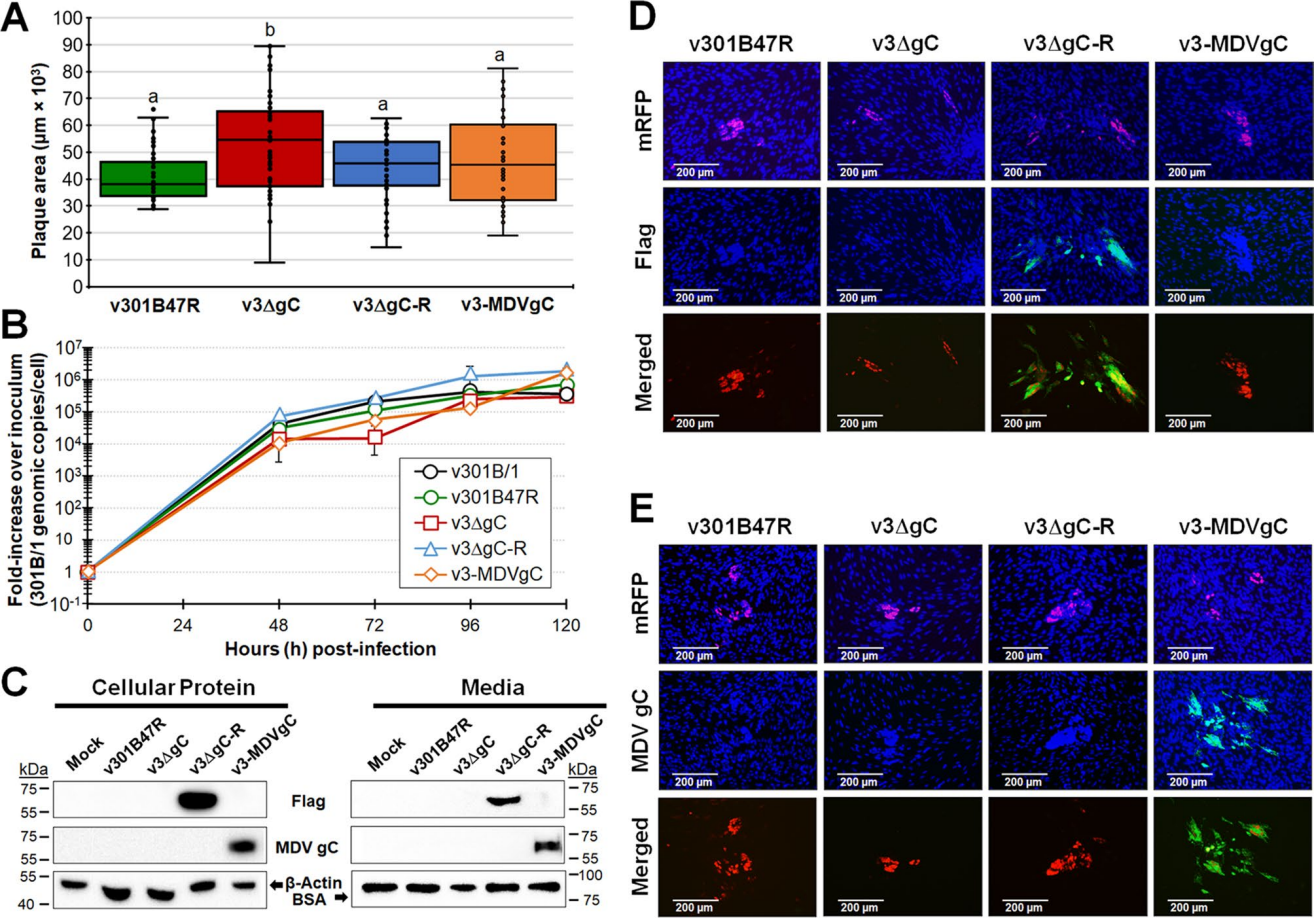
Replication and expression of proteins in cell culture. (**A**) Mean plaque areas for viruses reconstituted from r301B47R, r3ΔgC, r3ΔgC-R, and r3-MDVgC were measured and shown as box & whisker plots. Significant differences were determined using one-way ANOVA (*p* < 0.05, n = 200). Mean plaque areas with different letters are significantly different using LSD and Tukey’s post hoc tests (*p* ≤ 0.05). (**B**) Multi-step growth kinetics was used to measure virus replication in CEC cultures. The mean fold-change in viral DNA copies over the inoculum is shown for each virus and time point. There were no differences in virus growth (*p* > 0.05, two-way ANOVA, LSD, n = 3) (**C**) Western blotting to confirm 301B/1 gC and MDV gC expression. Both total cellular protein and infected cell culture media were used to detect 3 × Flag tagged 301B/1 gC and MDV gC. Anti-Flag M2 was used to detect 301B/1 gC, while anti-MDV gC A6 antibody was used to confirm MDV gC expression. For protein loading control, mouse anti-β-actin was used for total protein, while anti-BSA was used for infected cell media. (**D** and **E**) Expression of 301B/1 gC in v3ΔgC-R and MDV gC in v3-MDVgC. Representative plaques for all four viruses were stained with anti-Flag (**D**) or -MDV gC A6 (**E**) antibodies with goat anti-mouse-Alexa488 (green) as secondary antibody. Fluorescent expression of mRFP (red) was directly visualized and cells were counterstained with Hoechst 33342 to visualize nuclei. Only anti-Flag (**D**) or -MDV gC (**E**) and pUL47mRFP are shown in the merged images.

Following reconstitution of r301B/1 clones with UL44 removed (v3ΔgC) and replaced with Flag-tagged 301B/1 gC (r3ΔgC-R) or MDV gC (r3-MDVgC), we tested replication in CEC cultures using plaque size assays (Fig. 3A). Removal of 301B/1 gC resulted in significantly large plaque sizes, which is consistent with what is observed for MDV^17,18^. Adding 3 × Flag301B gC restored smaller plaque sizes (v3ΔgC-R), while adding MDV gC also restored smaller plaque sizes that were significantly different to v3ΔgC. However, virus growth kinetics measuring viral DNA replication in qPCR assays showed no significant differences (Fig. 3B). Figure 3C shows western blotting of total protein and media from infected cells using anti-MDV gC and -Flag antibodies. The rescued 301B/1 gC could be detected using the anti-Flag antibody in both cellular protein extracts and infected cell media, suggesting 301B/1 gC is also secreted as has been previously shown for MDV gC^17,33^. We also confirmed MDV gC expression in both infected cells and was in the media of infected cells. Immunofluorescence assays (IFA) were used to examine expression in cells and showed that Flag-tagged 301B/1 gC (Fig. 3D) and MDV gC proteins (Fig. 3E) were detected as expected. These results show that adding the 3 × Flag epitope to the N-terminus 301B/1 gC did not affect 301B/1 replication in cell culture and allowed us to identify its expression in vitro. Also, 301B/1 expressing MDV gC did not affect replication based on plaque size assays and MDV gC was expressed in v3-MDVgC.

### 301B/1 gC is required for interindividual spread

**Figure 4:**
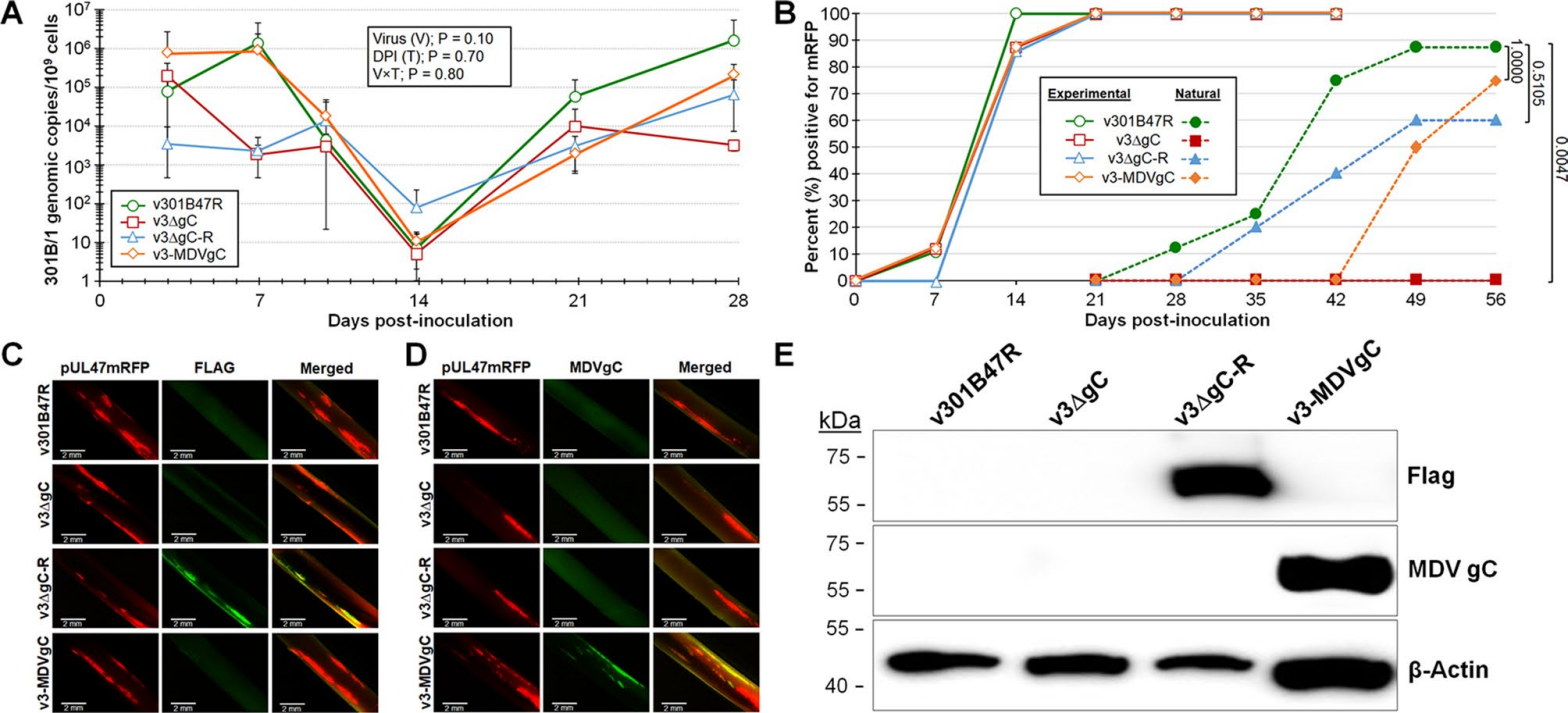
Replication and interindividual spread of r301B/1 viruses in chickens. Pure Columbian chickens were experimentally infected with v301B47R, v3ΔgC, v3ΔgC-R, or v3-MDVgC as described in the Materials and Methods for 56 days. (**A**) Replication was monitored in experimentally infected chickens by quantification of 301B/1 genomes in the blood over the first 4 weeks of infection. Shown is the mean 301B/1 genomic copies per 10^6^ blood cells ± standard error of means. No significant differences (*p* > 0.05, n = 109) were determined between all viruses at the same time point. (**B**) Quantitative analysis of the percent of birds positive for pUL47mRFP in FFs over the course of the experiment. Using Fisher’s exact test at *p* < 0.05, there was no significant difference in the total number of chickens positive for experimentally infected chickens with 100% positive by 21 days pi. No naïve contact chickens housed with v3ΔgC were naturally infected, while 88, 75, and 60% of contact chickens were naturally infected with v301B47R, v3-MDVgC, or vΔgC-R, respectively. Using Fisher’s exact test at *p* < 0.05, there was no significant difference between v3ΔgC-R (*p* = 0.5105) and v3-MDVgC (*p* = 1.0000) compared to v301B47R, while v3ΔgC was significantly different (*p* = 0.0047). (**C** and **D**) Feathers were plucked from v301B47R, v3ΔgC, v3ΔgC-R, and v3-MDVgC at 28 dpi, fixed, then stained using anti-Flag M2 (**C**) or anti- MDV gC (**D**) antibodies. FFs obtained from v3ΔgC-R-infected birds were positive for 3 × Flag301B gC, while FFs from v3-MDVgC -infected chickens were positive for MDV gC protein. (**E**) Western blot analysis for 3 × Flag301B and MDV gC in FFEs. Whole-cell protein lysates were collected from FFE cells scraped from infected FFs, electrophoresed through a 10% SDS-PAGE gel, transferred to nitrocellulose membranes, and probed for Flag or MDV gC as described in the Materials and Methods. Anti-β-actin antibody was used as internal cellular control.

To test our hypotheses that GaHV-3 gC, like MDV gC, would be required for interindividual spread in chickens, we tested our newly derived v3ΔgC using our experimental and natural infection model for interindividual spread. To do this, 8–10 chickens were inoculated with 10,000 PFU of each virus and housed with 6–10 uninfected chickens over the course of 8 weeks. Using qPCR assays to measure 301B/1 replication in the blood of experimentally infected chickens (Fig. 4A) and presence in FFs (Fig. 4B), no differences were seen between v301B47R, v3ΔgC, and v3ΔgC-R. However, when contact chickens were monitored for natural infection, no chickens housed with v3ΔgC-infected birds became infected compared to 88% and 60% of contact chickens were infected with v301B47R and v3ΔgC-R, respectively (Fig. 4B). Following termination, whole blood was collected from all contact chicken, serum was tested for anti-GaHV-3 antibodies using IFA and blood was used to measure 301B/1 viral DNA. It was confirmed all chickens negative for fluorescent FFs were also negative for anti-GaHV-3 antibodies and 301B/1 viral DNA in the blood (data not shown). IFA (Fig. 4C) and western blotting (Fig. 4E) were used to confirm the 3 × Flag epitope remained fused to the 301B/1 gC protein. With these results, we can conclude that 301B/1 gC is required for interindividual spread of the 301B/1 MD vaccine strain and the addition of a 3 × Flag epitope at the N-terminus of 301B/1 gC does not affect its function during interindividual spread.

### MDV gC can compensate for 301B/1 gC during 301B/1 interindividual spread

To test our second hypothesis that MDV gC would compensate for 301B/1 replication and transmission, we also tested v3-MDVgC in vivo. There was no difference in virus replication in the blood using qPCR assays (Fig. 4A) nor the ability to reach the FFs (Fig. 4B). Interestingly, v3-MDVgC was able to naturally infect chickens similar to v301B47R and v3ΔgC-R showing that MDV gC can compensate for 301B/1 in this essential function in vivo. IFA (Fig. 4D) and western blotting (Fig. 4E) were used to confirm MDV gC expression was maintained during replication in FFE cells. These results show that MDV gC can compensate for 301B/1 gC during 301B/1 MD vaccine strain interindividual spread in chickens.

## Discussion

In this report, we tested the importance of the alphaherpesvirus conserved gC protein for interindividual spread of the MD vaccine strain 301B/1. In addition, we tested whether MDV gC could compensate for 301B/1 gC in transmission and whether N-terminal tagging of 301B/1 gC would affect its function during interindividual spread. We were able to confidently conclude that 301B/1 gC is required for interindividual spread of 301B/1 virus and the addition of a 3 × Flag epitope at the N-terminus did not affect its function during transmission. We were also able to conclude that MDV gC can compensate 301B/1 gC during interindividual spread of 301B/1 virus. This data, combined with our former work on MDV^15,16^, suggests the essential role for the alphaherpesvirus conserved gC during interindividual spread is a conserved function of avian herpesviruses.

The exact role of MDV and 301B/1 gC during interindividual spread is not completely understood, but the absolute requirement during natural infection suggests it may be involved in virus-cell binding to cells. Homologs of gC perform multiple functions in vitro that include primary attachment of cell-free virus to proteoglycans on the surface of cells^2,3,34,35^ but is not required for specific interactions on cells where glycoprotein (gD) normally performs this function^36,37^. For HSV-1, gD binds to the herpesvirus entry mediator (HVEM), nectin-1, nectin-2, or modified heparin sulfate on the surface of cells providing a mechanism for cell tropic binding^38^ and it is believed gD of other members of the *Alphaherpesvirinae* perform similar functions. However, formerly we have shown that gD is not required for MDV interindividual spread^15^. The absolute requirement for MDV, and now 301B/1, gC in this process suggests it plays a more direct role in binding to cells during MDV and 301B/1 natural infection. Based on the ability of 301B/1 to naturally infect chickens when expressing 301B/1 or MDV gC and the close sequence homology (72.655% protein identity) between the two proteins (Fig. 1E), both proteins may target the same cellular protein and cell type to initiate infection. We are currently performing studies to elucidate potential binding partners for gC.

Currently, most MD vaccines do not transmit efficiently in chickens and thus cannot compete with virulent virus that does spread efficiently. Read et al.^39^ showed that current MD vaccines can enhance transmission of virulent MDV in the field, possibly because they are unable to block infection of chickens and shedding of virus. The fact that 301B/1 is as effective as traditional vaccine strains^23^ and is able to efficiently transmit in chickens suggests this vaccine strain may better protect unvaccinated or “missed” chickens in a flock and potentially compete with virulent MDV for replication in the skin. On top of that, swapping MDV gC for 301B/1 will most likely provide better immunogenic responses to MDV as gC is a major antigenic target against MDV^40^ and could increase its protective index.

Another important result in this report was that both 301B/1 and MDV gC proteins were secreted into the infected culture cell media suggesting alternative splicing of 301B/1 and MDV gC occurs in 301B/1 as has been shown for MDV gC during MDV cell culture propagation^17^. Further analysis on the splicing of the gC mRNA during 301B/1 replication is warranted; however, this data suggests the splicing of gC transcripts is also conserved in 301B/1 and the mechanism of gC transcript splicing, such as ICP27 and pUL47^32,41^, may also be conserved among the avian herpesviruses.

In summary, our results support our hypothesis that the absolute requirement of gC during interindividual spread is conserved among alphaherpesviruses. This report extends our work on MDV gC requirement to the MD vaccine strain 301B/1, and we can conclude at least for some avian herpesviruses with similar pathogeneses, that the functional importance of gC during interindividual spread is conserved. Further studies are warranted to determine whether gC homologs of other alphaherpesvirus are required for natural infection, although there are limited natural animal models to perform such studies making the avian herpesvirus models important for understanding conserved herpesvirus genes during natural infections.

## Materials and methods

### Cell culture and cells

All cells were maintained at 38 °C in a humidified atmosphere of 5% CO_2_. Chicken embryo cells (CEC) were prepared from 10 to 11-day-old specific-pathogen-free (SPF) embryos obtained from the University of Illinois at Urbana-Champaign (UIUC) Poultry Farm following standard methods^42^. Briefly, primary CEC cultures were seeded in growth medium consisting of Medium 199 (Cellgro, Corning, NY, USA) supplemented with 10% tryptose-phosphate broth (TPB), 0.63% NaHCO_3_ solution, antibiotics (100 U/ml penicillin and 100 µg/ml streptomycin), and 4% fetal bovine serum (FBS). Confluent CEC cultures were maintained in Medium 199 supplemented with 7.5% TPB, 0.63% NaHCO_3_, 0.2% FBS, and antibiotics.

The chicken DF-1-Cre fibroblast cell line^43^ was cultivated in a 1:1 mixture of Leibovitz L-15 and McCoy 5A (LM) media (Gibco, Gaithersburg, MD, USA) supplemented with 10% FBS and antibiotics (100 U/ml penicillin and 100 µg/ml streptomycin), and maintained in 50 µg/ml Zeocin (Invitrogen, Carlsbad, CA).

### Generation of two-step red recombination shuttle vectors

To produce pEP-301BgC-in, 301B/1 UL44 was Gibson assembly cloned from r301B/1 BAC DNA into pcDNA3.1 using primers shown in Table 2. Briefly, 301B/1 UL44 was amplified by PCR using a set of primers encompassing the complete UL44 gene, gel purified, and cloned into the pcDNA3.1 TOPO vector (Life Technologies) using Gibson Assembly reaction mix (NEB) according to the manufacturer’s instructions to generate pc301BgC. Next, a 3 × Flag epitope was cloned into pc301BgC from a previously described r3 × Flag54 BAC clone^32^ using Gibson assembly to generate pc3 × Flag301BgC. Next, the *aphAI*-I-*Sce*I cassette was amplified from pEP-KanS2 using primers shown in Table 2 and inserted into pc3 × Flag301BgC using Gibson Assembly cloning to generate pEP-301BgC-in. All clones at each step were confirmed by PCR and DNA sequencing. For insertion of MDV gC (RB1B strain) into r301B/1, a previously described pEP-MDVgC-in shuttle vector was used^17^.

**Table 2:** Primers used for cloning and generation of shuttle vectors using Gibson assembly cloning.

Construct^a^	Direction^b^	Sequence (5′ → 3′)
pc301B gC	Vector For	ACATATTACTTTCGTCCGTCGGTAAGCCTATCCCTAACCCTCTCC
	Vector Rev	GACGCGTGCATGGGGAAAATTCCGAGCTCGGTACCAAGCTTAACTAG
	Insert For	AGCTTGGTACCGAGCTCGGAATTTTCCCCATGCACGCGTCACG
	Insert Rev	GGGTTAGGGATAGGCTTACCGACGGACGAAAGTAATATGTATTTTTTCCCGG
pc3 × Flag301B gC	Vector For	ACAAGGATGACGACGATAAGATTAACCCCGATCTAGCTACACCC
	Vector Rev	CCGTCATGATCCTTGTAATCGCTAGCGCTTAGGACGCG
	Insert For	GCCGCGTCCTAAGCGCTAGCGATTACAAGGATCATGACGGAGATTACAAGG
	Insert Rev	GTAGCTAGATCGGGGTTAATCTTATCGTCGTCATCCTTGTAATCGATGT
pEP-301BgC-in	Vector For	GGCATAGAAATATCATCAGCCGAATATTACTCC
	Vector Rev	CGATTACCCTGTTATCCCTAGCTGATGATATTTCTATGCCGCTTGAG
	Insert 1 For	GGCATAGAAATATCATCAGCTAGGGATAACAGGGTAATCGATTTATTCAACAAAG
	Insert 1 Rev	CCTGCAAAGACCTGTAACCAGCCAGTGTTACAACCAATTAACCAAT
	Insert 2 For	TAATTGGTTGTAACACTGGCTGGTTACAGGTCTTTGCAGGACCC
	Insert 2 Rev	TCATCTTGGAGTAATATTCGGCTGATGATATT

^a^Construct generated with the set of primers.

^b^Directionality of the primer and product produced for Gibson assembly cloning.

### Generation of r301B/1 clones

To create 301B/1 expressing fluorescent-tagged pUL47, the coding sequence of the monomeric red fluorescent protein (mRFP) gene was inserted in frame at the C-terminus of the 301B/1 UL47 ORF by two-step Red-mediated mutagenesis^44^ in an infectious BAC clone of 301B/1. Briefly, the mRFP-I-*Sce*I-*aphAI* cassette was amplified from pEP-mRFP-in^45^ using primers shown in Table 3 and used for mutagenesis in GS1783 *Escherichia coli* cells. Multiple integrates and resolved clones were screened by RFLP analysis, analytic PCR, and DNA sequencing using primers shown in Table 1.

**Table 3:** Primers used for generation of recombinant *Gallid alphaherpesvirus* 3 strain 301B/1.

Modification^a^	Direction^b^	Sequence (5′- 3′)^c^
UL47mRFP	Forward	**AGAAGATGCGAAGGAGGCGATCTTCAAAAAAACGGACCGG***ATGGCCTCCTCCGAGGACG*
	Reverse	***TCACCACGATCTGCACGCCGCTCCGTGCGCTTTTTTTTTA****CAAGGCGCCGGTGGAGTG*
ΔgC	Forward	**ATATACGCTCTCGGAGACGCGGCTCGCACG**CCAGCTGAAATATTTTCCCCTAGTTTGCGGTGACATTGAT*TAGGGATAACAGGGTAATCGATTT*
	Reverse	***TACAAGAGCTCGGGGCATATAATGAGCCAG***ATCAATGTCACCGCAAACTAGGGGAAAATATTTCAGCTGG*GCCAGTGTTACAACCAATTAACC*
ΔgC-R (3 × Flag301BgC)	Forward	**GGCTCGCACGCCAGCTGAAATATTTTCCC***CCCC*ATG*CACGCGTCACG*
	Reverse	***AATGAGCCAGATCAATGTCACCGCAAA***CTA*GACGGACGAAAGTAATATGTATTTTTTCCCG*
MDV gC	Forward	**ATATACGCTCTCGGAGACGCGGCTCGCACG***TATCTTCCCTCATGCTCACG*
	Reverse	***TACAAGAGCTCGGGGCATATAATGAGCCAG****CATAACAATGAGATTATAAT*

^a^Modification to the 301B/1 genome using two-step Red-mediated recombination.

^b^Directionality of the primer.

^c^Underlined sequence indicates start and stop codons for 301B/1 UL44 gene**.** *Italics indicate the template-binding region of the primers for PCR amplification with pEP-mRFP-in, pEP-KanS2, pEP-301BgC-in, or pEP-MDVgC-in.* Bold indicates unique upstream integration sequences. Bold italic indicates unique downstream integration sequences.

To create r3ΔgC, the coding sequence of 301B/1 UL44 (gC) was deleted from r301B47R. Briefly, the I-*Sce*I-*aphAI* cassette from pEP-KanS2 was amplified by PCR with Thermo Scientific Phusion Flash High-Fidelity PCR Master Mix using primers shown in Table 3 and used for mutagenesis in GS1783 *E. coli* cells. Following removal of UL44 in the r301B47R clone, 301B/1 gC with a 3 × Flag epitope inserted at the N-terminus after the predicted signal sequence, or MDV gC were inserted into r3ΔgC using two-step Red recombination. Briefly, 3 × Flag301B/1 gC or MDV gC were PCR amplified from pEP-301BgC-in or pEP-MDVgC-in, respectively, using primers shown in Table 3 and used for mutagenesis as described above. RFLP analysis, analytical PCR, and DNA sequencing confirmed all clones were correct. Primers used for MDV gC have been previously published^17,46,47^, while primers for sequencing 301B/1 gC are listed in Table 1.

r301B/1 s were reconstituted by transfecting DF-1-Cre cells with purified BAC DNA plus Lipofectamine 2000 (Invitrogen) using the manufacturers’ instructions as previously described^29^. Transfected DF-1-Cre cells were mixed with fresh primary CEC cultures until plaques formed, then further propagated in CEC cultures until virus stocks could be stored. All viruses were used at ≤ 5 passages for in vitro and in vivo studies.

### Immunofluorescence assays (IFA)

CEC cultures were infected with different r301B/1 viruses on sterile glass coverslips at 100 plaque-forming units (PFU) per well. At 5 days post-infection (p.i.), cells were fixed with PFA buffer (2% paraformaldehyde, 0.1% Triton X-100) for 15 min and then washed twice with PBS. Fixed coverslips were blocked in 10% neonatal calf serum and stained with anti-GaHV-3 chicken sera and goat anti-chicken IgY-Alexa Fluor 488 secondary antibody (Molecular Probes, Eugene, OR). To detect 3 × Flag301BgC, mouse anti-Flag M2 (Sigma-Aldrich) was used. Anti-gC monoclonal A6 (kindly provided by Jean-Francois Vautherot, INRA, Nouzilly, France) antibody^48^ was used to detected MDV gC expression. Anti-mouse Ig Alexa Fluor 488 (Molecular Probes, Eugene, OR) was used as secondary antibody for both anti-Flag and -MDV gC monoclonal antibodies. The virus plaques were observed using an EVOS FL Cell Imaging System (Thermo Fisher Scientific) and compiled using Adobe Photoshop version 21.0.1.

### Measurement of plaque areas

Plaque areas were measured in CEC cultures exactly as previously described^49^ using anti-GaHV-3 chicken sera and goat anti-chicken IgY-Alexa Fluor 488 secondary antibody (Molecular Probes, Eugene, OR). Digital images of 50 individual plaques were collected using an EVOS FL Cell Imaging System (Thermo Fisher Scientific) and compiled with Adobe Photoshop version 21.0.1. Plaque areas were measured using ImageJ^50^ version 1.53d software (http://imagej.nih.gov/ij). Box and Whisker plots were generated, and significant differences were determined using IBM SPSS Statistics Version 27 software.

### Viral growth kinetics in cell culture

To measure viral growth kinetics of viruses in cell culture, qPCR assays were used to measure the relative level of replication as previously described^23^. Briefly, CEC cultures were prepared in 6-well tissue culture plates and the next day inoculated with 100 PFU/well. Total DNA was collected from the inoculum and at 48, 72, 96, and 120 h following infection using the QIAamp DNA Mini Kit (Qiagen, Germantown, MD). Quantification of 301B/1 genomic copies in CEC cultures was performed using primers and probe previously described^23^ and were used in duplex PCR reactions with previously described primers and probes against chicken iNOS^51^. All qPCR assays were performed in an Applied Biosystems QuantStudio 3 Real-Time PCR System (Thermo Fisher Scientific) and the results were analyzed using the QuantStudio Design & Analysis Software v1.4.2 supplied by the manufacturer. The fold-increase in viral DNA copies over inoculum was used to determine differences in replication.

### Western blot analysis

Western blot analyses were performed as previously described^48^. Total protein was collected from infected CEC cultures or scraped from FFE previously described^18^. In some experiments, infected CEC culture media was collected to detected secreted proteins. To detect the relative level of 301B/1 infection, mouse anti-GaHV-3 Y5.9^52^ was used at 1:500 dilution to detect GaHV-3 specific gB. To detect mRFP tagged 301B/1 pUL47, rabbit anti-mRFP antibody (ab62341; Abcam) was used with secondary sheep anti-rabbit IgG (H + L)-HRP conjugate (A16172; Life Technologies, Inc.). To detect MDV gC, monoclonal antibody A6 was used at a 1:500 dilution. To detect 3 × Flag tagged 301B/1 gC, anti-Flag M2 was used and for protein loading control, mouse anti-β-actin (ACTNO5; Abcam, Cambridge, MA) was used at their recommended dilutions. Anti-bovine serum albumin (BSA, Thermo Fisher Scientific) mAb was used at its recommended dilution as a loading control for infected cell media. Secondary anti-mouse IgG peroxidase conjugate was purchased from GE Healthcare (Piscataway, NJ) and used for mouse monoclonal antibodies. The SuperSignal West Pico Chemiluminescent Substrate kit from Thermo Fischer Scientific (Rockford, IL) was used to detect antigens using the manufacturer’s instructions.

### Ethics statement

All animal work was conducted according to national regulations and ARRIVE guidelines. The animal care facilities and programs of UIUC meet the requirements of the law (89 –544, 91–579, 94 –276) and NIH regulations on laboratory animals and are compliant with the Animal Welfare Act, PL 279. UIUC and the College of Veterinary Medicine at UIUC are accredited by the Association for Assessment and Accreditation of Laboratory Animal Care (AAALAC). All experimental procedures were conducted in compliance with approval of UIUC’s Institutional Animal Care and Use Committee (IACUC).

### Animal experiments

Pure Columbian (PC) chickens were obtained from the UIUC Poultry Farm (Urbana, IL) and were from MD-vaccinated parents; therefore, considered to be maternal antibody positive. To test replication of v301B47R, 7-day old chicks (n = 10) were experimentally infected by intra-abdominal inoculation of 4000 PFU for v301B47R and housed with another ten chickens that were left uninfected to act as contacts to determine whether v301B47R can naturally infect naïve chickens by interindividual spread. To test the ability of v301B47R, v3ΔgC, v3ΔgC-R, v3-MDVgC to replicate and interindividual spread in chickens, 3-day old PC chicks (n = 8–10/group) were inoculated with 10,000 PFU with each respective virus and housed in separate rooms. To test natural infection through interindividual spread, 6–8 age-match, naïve contact chickens were housed experimentally infected chickens for eight weeks. Water and food were provided ad libitum for all animal experiments.

### DNA extraction from blood cells and qPCR assays

Whole blood was obtained by wing-vein puncture and DNA was extracted using the E.Z. 96 blood DNA kit from Omega Bio-tek, Inc. (Norcross, GA) as previously described^15^. Quantification of 301B/1 genomic copies in the blood using qPCR was performed using primers and probe previously described^23^ and were used in duplex PCR reactions with previously described primers and probes against chicken iNOS^51^. All qPCR assays were performed in an Applied Biosystems QuantStudio 3 Real-Time PCR System (Thermo Fisher Scientific) and the results were analyzed using the QuantStudio Design & Analysis Software v1.4.2 supplied by the manufacturer. The final viral loads were obtained after normalizing with chicken iNOS used as an internal control gene.

### Monitoring v301B47R and its derivatives in feather follicles (FFs)

To track the time at which each r301B47R or its derivatives reached the FFs, two flight feathers were plucked from the right and left wings (4 total) of inoculated birds weekly and pUL47mRFP expression was examined using a Leica M205 FCA fluorescent stereomicroscope with a Leica DFC7000T digital color microscope camera (Leica Microsystems, Inc., Buffalo Grove, IL).

### IFA of feather follicles (FFs)

Whole feathers were plucked from chickens infected with different r301B/1s and the FFs were fixed using PFA buffer, washed twice with PBS, and then blocked in 10% neonatal calf serum (Sigma-Aldrich). Fixed FFs were stained with primary mouse anti-Flag M2 (Sigma-Aldrich) or anti-MDV gC A6^48^ antibodies with anti-mouse Ig Alexa Fluor 488 (Molecular Probes, Eugene, OR) used as secondary antibody. The Leica M205 FCA fluorescent stereomicroscope with a Leica DFC7000T digital color microscope camera (Leica Microsystems, Inc., Buffalo Grove, IL) was used to analyze stained FFEs. All images were compiled using Adobe Photoshop version 21.0.1.

### Statistical analyses

Statistical analyses were performed using IBM SPSS Statistics Version 27 software (SPSS Inc., USA). Plaque size assays were analyzed with Student’s *t* tests and one-way analysis of variance (ANOVA), virus was included as fixed effect and the plaque size was used as dependent variable. The normalized data of viral replication (qPCR) were analyzed using two-way ANOVA followed by LSD and Tukey’s post hoc tests; virus (V) and time (T) and all possible interactions (V × T) were used as fixed effects, and the genomic copies as dependent variable. Fisher’s exact test was used for infection and transmission experiments. Statistical significance was declared at *p* < 0.05 and the mean tests experiments associated with significant interaction (*p* < 0.05) were separated with Tukey’s test.
